# Development and clinical application of a preimplantation genetic testing for monogenic disease (PGT-M) for beta thalassemia in Vietnam

**DOI:** 10.1007/s10815-020-02006-y

**Published:** 2020-11-20

**Authors:** Anh Dao Mai, Gary L. Harton, Vinh Nguyen Quang, Huynh Nguyen Van, Nhung Hoang Thi, Nga Pham Thuy, Thu Hien Le Thi, Duc Nguyen Minh, Quan Tran Quoc

**Affiliations:** 1 Genetic Testing Service Joint Stock Company, 249A Thuy Khue street, Tay Ho district, Hanoi, Vietnam; 2 PerkinElmer Health Sciences Australia, 40 West Thebarton Rd., Thebarton, SA 5031 Australia; 3 Reproductive Health Assistant and Andrology Department, Hanoi Obstetrics &Gynecology Hospital, 929 La Thanh street, Ngoc Khanh ward, Ba Dinh district, Hanoi, Vietnam; 4 Andrology and Fertility Hospital of Hanoi, 431 Tam Trinh street, Hoang Mai district, Hanoi, Vietnam

**Keywords:** Preimplantation genetic testing for monogenic diseases (PGT-M), Preimplantation genetic testing for aneuploidy (PGT-A), Embryo biopsy, Aneuploidy, Beta thalassemia

## Abstract

**Purpose:**

The purpose of this research is to study the clinical outcomes using a next-generation sequencing-based protocol allowing for simultaneous testing of mutations in the beta thalassemia (HBB) gene, including single nucleotide polymorphism (SNP) markers for PGT-M along with low-pass whole genome analysis of chromosome aneuploidies for PGT-A.

**Methods:**

A combined PGT-M (thalassemia) plus PGT-A system was developed for patients undergoing IVF in Vietnam. Here we developed a system for testing numerous thalassemia mutations plus SNP-based testing for backup mutation analysis and contamination control using next-generation sequencing (NGS). Low -pass next-generation sequencing was used to assess aneuploidy in some of the clinical PGT cases. Patients underwent IVF followed by embryo biopsy at the blastocyst stage for combined PGT-A/M.

**Results:**

Two cases have completed the entire process including transfer of embryos, while a further nine cases have completed the IVF and PGT-M/A analysis but have not completed embryo transfer. In the two cases with embryo transfer, both patients achieved pregnancy with an unaffected, euploid embryo confirmed through prenatal diagnosis. In the further nine cases, 39 embryos were biopsied and all passed QC for amplification. There were 8 unaffected embryos, 31 carrier embryos, and 11 affected embryos. A subset of 24 embryos also had PGT-A analysis with 22 euploid embryos and 2 aneuploid embryos.

**Conclusions:**

Here we report the development and clinical application of a combined PGT-M for HBB and PGT-A for gross chromosome aneuploidies from 11 patients with detailed laboratory findings along with 2 cases that have completed embryo transfer.

## Introduction

Preimplantation genetic testing for monogenic diseases (PGT-M), formerly termed preimplantation genetic diagnosis (PGD), was the first genetic test offered on preimplantation embryos [1] in 1990 with the first babies born in 1991. Generally, the test requires in vitro fertilization (IVF) followed by embryo biopsy using fine needles and/or chemicals (i.e., acid Tyrode) to breach the zona pellucida of the embryo. After the hole is created, one or two single blastomeres from the developing embryo can be removed for genetic testing, allowing the rest of the embryo to continue to grow for 2 more days in culture while the testing was carried out prior to the transfer of an unaffected embryo [2].

For the molecular laboratory, having multiple cells to work with removes some of the potential issues that surrounded early attempts at single-cell biopsy including reductions in allele dropout (ADO) and preferential amplification (PA). Allele dropout arises in the early stages of amplification by polymerase chain reaction (PCR), where one allele of a heterozygote is not primed and products for this allele are not created. In a heterozygote cell, this means that one allele will be missed which can lead to misdiagnosis as a homozygous embryo [3]. Preferential amplification is similar to ADO except that some product is made for one allele while more product is made for the other allele. Again, depending on the downstream method of analysis chosen, this can lead to errant results and potential misdiagnosis. Dreesen and colleagues have published a thorough review of misdiagnosis during PGT-M (PGD) [4].

New methods have been developed to eliminate or at least minimize some of the issues noted above. Many groups started adding linked markers, typically short tandem repeat (STR) DNA markers that were found inside of the gene or closely linked to the gene of interest [5]. Utilizing these tightly linked markers allows for a secondary analysis of inheritance of the gene in question and also allows the laboratory to track contamination of DNA from exogenous sources including maternal, paternal, IVF lab staff, and molecular lab staff. This technique continues to be the predominant tool for PGT-M today.

In 2010, Handyside and colleagues announced a new method to diagnose preimplantation embryos for almost any disease (assuming the gene has been located). This technology, called Karyomapping, utilizes a commercially available single nucleotide polymorphism (SNP) array that contains approximately 300,000 unique SNPs across the entire genome [6]. By assessing which parent contributed each part of the chromosome, Karyomapping can discern the inheritance of the region of the chromosome where a gene of interest is located. Karyomapping also allows the user to look at some aneuploidies present in the early embryo but not all aneuploidies are accurately detected. In addition, Karyomapping might be considered to be more expensive than traditional PCR-based PGT-M testing and requires multiple samples from family members to establish a reference before embryo testing can commence, which is not always feasible.

In addition to Karyomapping, a second commercial system from Agilent allows for simultaneous analysis of single gene defects along with screening of common aneuploidies found in early human embryos. This technology is called OnePGT and utilizes whole genome amplification using multiple displacement amplification (MDA) followed by next-generation sequencing for analysis of specific single-gene disorders and/or aneuploidy detection from a single bar-coded sample. In one published study using this platform, good concordance for both PGT-M and PGT-A was reported from leftover WGA material and/or secondary biopsy of supernumerary embryos [7]. In this study, approximately 15% of the samples could not be analyzed due to biologic interference and/or crossing over in or near the mutation site. For this system, the NGS must be performed on Illumina sequencing technology platforms (NextSeq or HiSeq2500) and require 16 × 10^6^ reads per sample for accurate PGT-M analysis. These sequencers are not typically found in PGT labs globally and the requirement for so many sequencing reads leads to low throughput of the system allowing only 18–24 samples per run [8].

The Masset study noted above utilized previously analyzed embryos to assess the concordance between OnePGT from Agilent and conventional assessment tools for PGT-M mainly PCR with STR analysis. The study presented here shows prospective analysis of PGT-M samples from 12 patients that carry thalassemia. Ultimately, there are a number of methods available to assess the gene mutation and chromosomal status of human embryos, each with pros and cons that need to be assessed by the genetics laboratory prior to utilization.

The target sequence enrichment (TSE) protocol aims to minimize many of the issues noted above offering specific mutation testing along with confirmatory single nucleotide polymorphisms (SNPs) and/or STRs analysis. In the TSE workflow, specific PCR primers are designed to assess the genetic mutation(s) of interest in the couple along with linked STR or SNP markers for contamination control and/or confirmatory analysis of disease status. These primer sets are added to the initial master mix for the WGA step using the DOPlify® protocol followed by thermal cycling according to manufacturer’s instructions. Once the initial WGA PCR is complete, an aliquot of the WGA product is removed and a secondary PCR is performed with either the same primer mix or a nested set of primers specific for the mutation and linked markers. This TSE/WGA product is then prepared for sequencing using any number of library preparation systems, followed by next-generation sequencing and analysis using PG-Find™ software for aneuploidy detection and open-source software for analysis of the mutation site and linked makers.

The TSE protocol also aims to reduce or eliminate ADO and PA in samples by reliably amplifying the targeted DNA sequences while preserving genome-wide amplification simultaneously. As Warren and colleagues showed, the use of the TSE protocol was superior to gene-specific PCR and combining WGA with primers spiked in for the DNA sequences of interest [9]. Here they showed that the TSE protocol had no ADO, compared to PCR primers only (which would not allow for PGT-A to be carried out) and to WGA with primers spiked in.

**Fig. 1:**
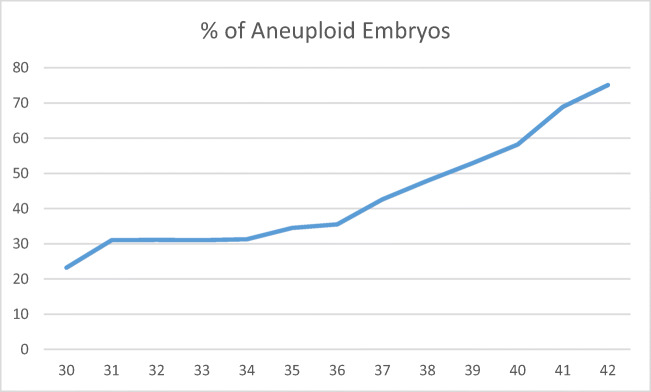
Adapted from Franasiak et al. 2014. Percentage (%) of aneuploid embryos by single year patient age in a large cohort of over 15,000 consecutive trophectoderm biopsies evaluated by preimplantation genetic testing for aneuploidy

While thousands of IVF cycles with PGT-M have been performed worldwide since the first attempts in 1992 [10], aneuploidy during PGT-M cycles remains a constant issue. In the article by DeRycke, the authors report on over 17,000 PGT-M cycles with a clinical pregnancy rate per oocyte retrieval of 21% and a clinical pregnancy rate per embryo transfer of 29%. In theory, these are fertile patients undergoing IVF to access PGT-M testing on their embryos before transfer. In a study looking at aneuploidy rates from infertility patients and broken down in 1 year increments [11], the euploid/aneuploid ratio for a 35-year-old was 65.5/34.5%, and for a 42-year-old that ratio was 24.9/75.1% (Fig. 1). Although a PGT-M result may indicate that an embryo is unaffected by a monogenic disease, standard PGT-M methods are unable to assess aneuploidy. Depending on the age of the female patient, somewhere between 44% and 75% of these transferred embryos may be aneuploid. A robust method combining WGA along with gene-specific primers in a single reaction to allow both aneuploidy detection (PGT-A) and monogenic disease detection (PGT-M) would be a great leap forward for patients undergoing PGT-M clinically.

Preimplantation genetic testing for aneuploidy (PGT-A) aims to test all embryos in an IVF cycle for aneuploidy and offer selective transfer of euploid embryos, those carrying the correct number of chromosomes. A number of studies and randomized clinical trials (RCT) have shown the benefit of PGT-A in improving pregnancy rates [12–14], decreasing pregnancy loss rates [15], and improving the time to pregnancy [16].

To date, combining PGT-A and PGT-M has proved to be difficult without some concessions from either test. Many labs perform combined PGT-A and PGT-M by following their normal WGA methods for PGT-A to a certain point and then split the WGA into 2 different samples and process each of them separately (personal communication). This system usually creates enough reads for PGT-A analysis and enough PCR product for downstream PGT-M testing; however, splitting the WGA products into separate aliquots creates a few obvious issues, including the chance for sample switches/failed amplification, preferential amplification, and increased allele drop out (ADO) rates. In addition, if the lab wants to use next-generation sequencing (NGS) technology for all downstream analysis, then this splitting typically creates samples with different bar codes, so the sample would need to be analyzed separately for PGT-A and PGT-M, which adds to the cost.

Thalassemia is a set of disorders that are inherited from parents to offspring. Patients with thalassemia create an abnormal form or an inadequate amount of hemoglobin, the protein found in red blood cells that carries oxygen throughout the body. Hemoglobin is a complex protein made up of alpha and beta globin chains. Alpha thalassemia occurs when there is a mutation in the gene that encodes the alpha globin gene and beta thalassemia occurs when there is a mutation in the beta globin gene. A patient with thalassemia can have varying severity of anemia. Thalassemia affects about 1.5% of the global population and 1.5% of Vietnamese population are carriers of beta thalassemia [17, 18].

Couples who are both beta thalassemia carriers usually turn to IVF with PGT-M to test for unaffected offspring. In the ESHRE PGD Consortium review of the first 10 years of data collection, testing for any of the thalassemia mutations represents one of the top 3 indications globally for PGT-M testing with over 500 cases reported within the first 10 data collection manuscripts [19]. Only cystic fibrosis and Duchenne muscular dystrophy are more commonly performed tests in PGT-M. As mentioned previously, PCR-based gene mutation analysis with STR linkage analysis is a well-known solution for PGT-M but allele dropout, STR mutation, and stutter peaks are still major problems.

Here we describe a method allowing for linkage-based analysis of the beta thalassemia mutation using SNP haplotyping along with detailed analysis of aneuploidy in all 23 chromosomes simultaneously. This method uses whole genome amplification (WGA) to allow low-pass sequencing coverage to detect aneuploidy down to approximately 10 MB in size along with target sequence enrichment (TSE) to specifically and reliably amplify the SNP set for each specific patient set. This method allows for PGT-M with no allele dropout or preferential amplification for beta thalassemia and PGT-A analysis all in a single tube and in a single economical sequencing run.

In addition, this technique takes advantage of a few improvements in the embryology laboratory that have been developed over the past decade or so. This includes newly developed embryo culture media that make growth to the blastocyst stage of embryo development routine. This is an important development because it allows for an initial selection of embryos and allows for biopsy of 3–5 cells from each embryo rather than a single cell from an earlier stage embryo. In addition, most embryology laboratories globally are now able to utilize vitrification for cryopreservation of embryos during analysis which offers nearly unlimited time to the genetics laboratory to perform testing and reporting. These improvements in the embryology laboratory have certainly led to better and more accurate results in the genetics laboratory.

## Materials and methods

In order to develop a nearly universal method to offer PGT-M for beta thalassemia with PGT-A, a set of unique single nucleotide polymorphism (SNP) markers unique to the Vietnamese population were curated by assessing previous published reports and/or searching public databases of known single nucleotide polymorphisms. Polymerase chain reaction (PCR) primers specific for a number of beta thalassemia gene mutations and the developed SNP markers were designed and validated ahead of clinical use by utilizing genomic DNA of known beta thalassemia carriers diluted to 5–20 pg/ul in the designed WGA/TSE protocol to assess amplification success rates and allele dropout across the entire platform. During validation, the mutation primer sets and SNP markers were 100% concordant with the expected genotype of the DNA (unpublished).

A cycle of in vitro fertilization (IVF) was offered, with growth of the embryos to the blastocyst stage followed by trophectoderm biopsy in order to offer simultaneous analysis of the mutations in each family plus the unique SNP markers. Patients were counseled at the IVF center(s) about the IVF procedure/PGT-M procedure prior to stimulation and consented verbally to the plan detailed below. No IRB approval was obtained for this case report as the subjects were not part of a clinical trial or any other study. No identifying details of the patient(s), their embryos, or their IVF cycles have been shared in this manuscript; in addition, the actual embryo number designations from the IVF cycles have been removed and all embryos, no matter their original designation from the IVF cycle, have been lettered A-X, where X is the total number of embryos biopsied during each cycle. According to the common rule 45 CFR 46.101(b) [4], exemptions from IRB include “research, involving the collection or study of existing data, documents, records, pathologic specimens, if these sources are publicly available or if the information is recorded by the investigator in such manner that subjects cannot be identified, directly or through identifiers linked to subjects.”

Briefly, the patient’s ovaries were stimulated using gonadotropins to produce multiple mature oocytes which were retrieved under ultrasound guidance. Mature oocytes were inseminated using intracytoplasmic sperm injection (ICSI) to minimize the risk of paternal contamination of the biopsy samples with extraneous sperm. Normally fertilized embryos were cultured for 5 to 7 days until they reached the blastocyst stage where they were biopsied using laser-assisted hatching followed by removal of 4–6 cells from the trophectoderm layer of the blastocyst. Following the biopsy, all blastocysts were vitrified and cryopreserved embryos were held on liquid nitrogen (approximately – 200 °C) while awaiting the results of the genetic test.

The biopsied material was washed and placed into individual sterile 0.2-ml PCR tubes labeled with the female patient’s initials and embryo number. The specimens were shipped to the genetics laboratory on dry ice, and upon arrival in the genetics laboratory, the samples were accessioned and subjected to whole genome amplification using DOPlify (PerkinElmer Inc. Waltham, MA), following the target sequence enrichment (TSE) protocol (PerkinElmer). The whole genome amplification protocol with TSE involves an initial enzyme-based cell lysis procedure to facilitate degradation of the cell membranes and make the DNA accessible for amplification. This is followed by an initial denaturation step (5 min at 95 °C) and a series of low-stringency cycles (8 cycles of 98 °C for 20 s, 25 °C for 1 min 30 s, ramping to 72 °C at 0.25 °C/s, 72 °C for 1 min). The slow temperature ramping during the annealing step of the low-stringency cycles allows the whole genome amplification degenerate oligonucleotide primer to bind at regular intervals across the genome, creating an array of fragments representative of the initial DNA template. After the low-stringency cycles of PCR, the WGA reaction was supplemented with a 10-μM pool of mutation and SNP-specific primers to facilitate enrichment of the beta thalassemia gene mutations and identified SNP locations. Following the addition of these primers, 21 cycles of high-stringency PCR were completed (98 °C for 20 s, 58 °C for 1 min, 72 °C for 1 min) followed by a final extension of 1 min at 72 °C. The high-stringency PCR conditions enable amplification of both the target sequences and the whole genome fragments created during the low-stringency PCR cycles.

For these experiments, SNP markers located within 300 kb of the HBB gene and meeting the requirement of heterozygosity > 0.3 were chosen. A full listing of the SNP markers used for each patient with full clinical outcomes can be found by reviewing Table 1 and Table 2.

**Table 1:**
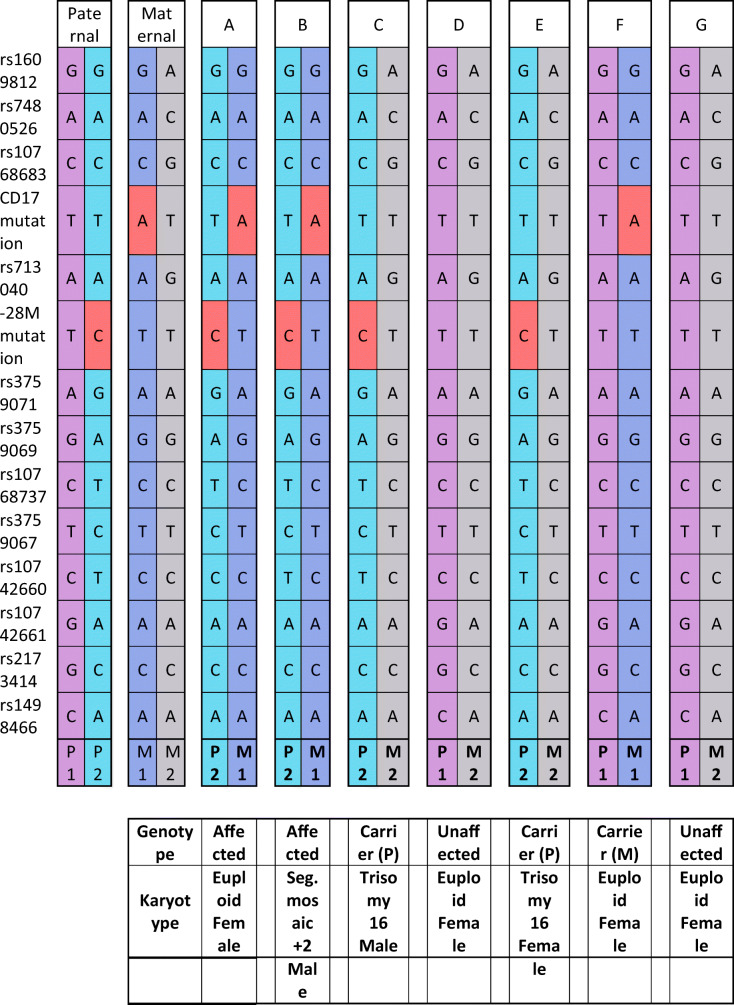
HBB mutation and SNPs genotyping results for case 1.

Legend: Genotype of parents and embryos assessing a CD17 mutation and − 28 M mutation of the HBB gene plus 12 SNPs closely associated with the mutation. P = paternal, M = maternal, 1 = allele 1, 2 = allele 2, red block = mutation, pink/purple/Gy = no mutation. Parental haplotypes represented by “P1/P2” meaning paternal haplotype 1 and paternal haplotype 2; same for maternal haplotypes are labeled and color coded for ease of interpretation

**Table 2:**
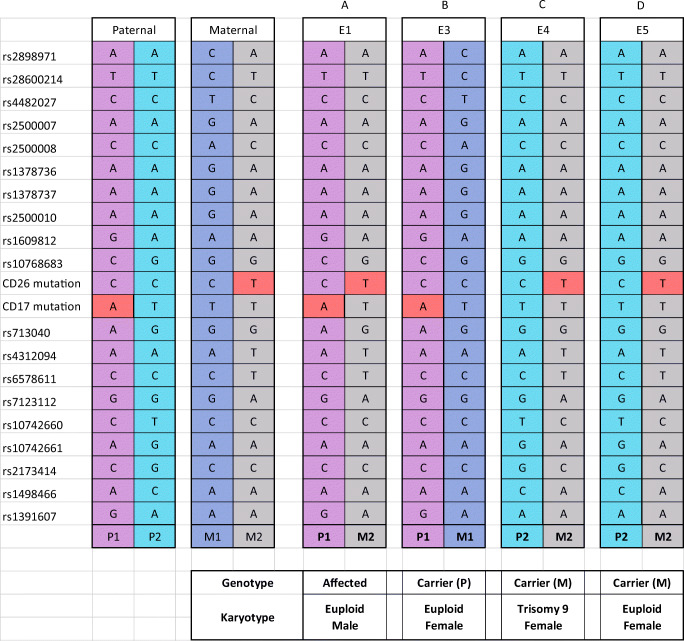
HBB mutation and SNPs genotyping results for case 2.

Legend: Genotype of parents and embryos assessing a CD17 mutation and CD26 mutation of the HBB gene plus 19 SNPs closely associated with the mutation. P = paternal, M = maternal, 1 = allele 1, 2 = allele 2, red block = mutation, pink/purple/Gy = no mutation. Parental haplotypes represented by “P1/P2” meaning paternal haplotype 1 and paternal haplotype 2; same for maternal haplotypes are labeled and color coded for ease of interpretation

Following the initial amplification, a secondary target enrichment PCR was performed using the WGA product as template to generate additional amplified product of the HBB and SNP target regions. This helps to ensure there is sufficient breadth and depth of coverage to enable mutation detection and SNP analysis. This secondary enrichment PCR reaction included 10 μl Platinum SuperFi PCR Master Mix (ThermoFisher Scientific, Waltham, MA, USA), 2 μl WGA products (4-10 ng), 3 μl primer mix (10 μM pool of the HBB and SNP primers), and 5 μl H_2_O (for the total of 20ul). The PCR program was as follows: 5 min at 95 °C, 35 cycles of 30 s at 95 °C, 30 s at 58 °C, 45 s at 72 °C, and a final extension of 15 min at 72 °C. Once this PCR is completed, an aliquot of the secondary PCR is spiked back into the initial WGA product, typically at 1:10 to 1:20 concentrations. This secondary PCR creates an extra procedure with the sample that could lead to sample mix-ups detailed above. The target-enriched PCR product was pooled back into the initial WGA product to enable a single library preparation reaction for each sample to be performed with a single unique index.

Pooling the target-enriched PCR product with the WGA product before library preparation and sequencing allows both copy number detection and target region analysis to be performed simultaneously and aids in sample tracking. Libraries from the pool of enriched PCR product and WGA product were prepared using the Nextera XT DNA library Prep kit (Illumina, Inc., San Diego, CA USA) (ratio WGA product: enriched PCR product = 10:1).

Next-generation sequencing (NGS) was performed on the Illumina MiSeq Instrument using the MiSeq v3 reagent kit and 1 × 76 cycles as per the DOPlify® kit manufacturer’s recommendation. Demultiplexing and sequence alignment to hg19 was completed with the MiSeq Reporter Software (Illumina). Aligned data was analyzed for chromosome copy number variations using both the Nexus Copy Number Software (BioDiscovery, Los Angeles, CA, USA) and the PG-Find™ software (PerkinElmer, Waltham, MA). Miseq Reporter (Illumina) software was used for mutation detection and SNP calling. Only heterozygous SNPs of the parents were used for linkage analysis.

Following analysis and reporting of the results, the patient was counseled by the clinical staff and a decision on which embryo to transfer was decided. Following warming of the vitrified blastocyst using standard protocols, the blastocyst was allowed to re-expand for approximately 1 h prior to transfer in a controlled endometrial development (CED) cycle. Clinical follow-up on pregnancies was guided by the referring IVF center including review of the need for follow-up prenatal testing by amniocentesis.

## Results

So far, two cases have been completed through embryo transfer following IVF and PGT-M/-A. For the first couple, 7 blastocysts were available for biopsy and testing and for the second couple, 5 blastocysts were available for biopsy and testing. Following WGA of the first two cases, it was confirmed that one sample out of 12 failed to amplify (91.7% amplification efficiency).

In each case, a single embryo was chosen for transfer based on the results of the specific single gene defect test for mutations in the HBB gene as well as aneuploidy results based on next-generation sequencing (NGS)–based analysis of aneuploidy in all 24 chromosomes. In addition, 9 further cases have been through IVF and/or PGT-M/-A using the same system as detailed above. At the time of this writing, we are awaiting further information from our IVF clinic partners on which embryo(s) have been transferred and further clinical outcomes.

**Fig. 2:**
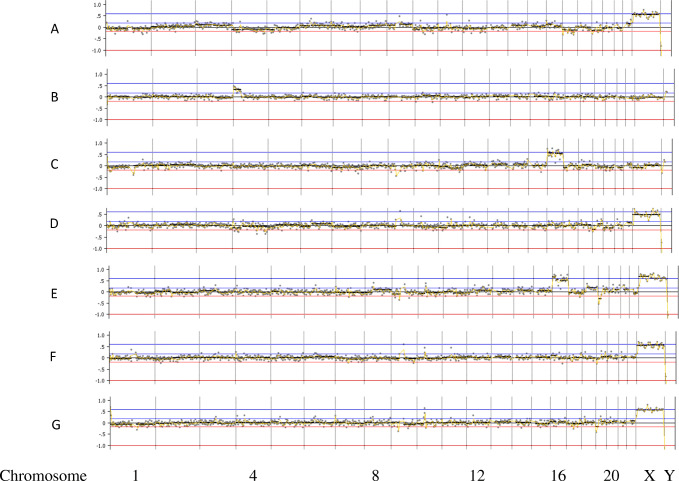
PGT-A results for case number 1. Legend: PGT-A results for case number 1. X-axis is chromosome number (1-22, X, Y); Y-axis is gain (+) and loss (−) following WGA using DOPlify, PG-Seq, and Nexus copy number software. Green dots are individual bin calls from NGS signifying the expected copy number of each bin. Black line is software “call” for gains, losses, or correct count of chromosomes

In case 1, seven embryos were biopsied at the blastocyst stage and all seven embryos showed amplification following WGA and were analyzed for mutations in the HBB gene. In this case, the paternal partner carried a CD17 mutation and the maternal partner carried a − 28 M mutation in the HBB gene. In all, 12 other SNPs in and around the HBB gene were also analyzed to help confirm diagnosis and control for contamination from exogenous DNA sources. Here, there were two embryos diagnosed as affected (A and B), two embryos that were diagnosed as carriers of the paternal HBB mutation (C and E), one embryo that was diagnosed as a carrier of the maternal HBB mutation (F), and two embryos that were diagnosed as unaffected (D and G). The mutation and SNP calls can be seen in Table 1. The same 7 embryos were also tested for aneuploidy across all 24 chromosomes with 4 euploid embryos (A, D, F, and G) and three aneuploid embryos (B-mosaic 4p gain, C-trisomy 16, E-trisomy 16). In this case, embryo F was chosen for transfer and resulted in a singleton pregnancy. The PGT-A results can be seen in Fig. 2. Amniocentesis was performed on the resulting pregnancy and showed a normal female result with the correct mutation call at the HBB locus as well.

**Fig. 3:**
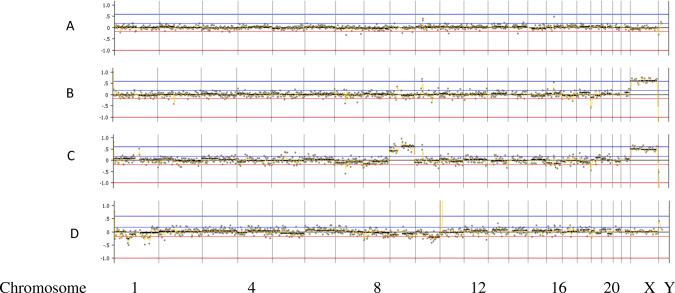
PGT-A results for case number 2. Legend: PGT-A results for case number 1. X-axis is chromosome number (1-22, X, Y); Y-axis is gain (+) and loss (−) following WGA using DOPlify, PG-Seq, and Nexus copy number software. Green dots are individual bin calls from NGS signifying the expected copy number of each bin. Black line is software “call” for gains, losses, or correct count of chromosomes

In case 2, five embryos were biopsied at the blastocyst stage and four of the five embryos showed amplification following WGA and were analyzed for mutations in the HBB gene. In this case, the paternal partner carried a CD26 mutation and the maternal partner carried a CD17 mutation in the HBB gene. In all, 19 other SNPs in and around the HBB gene were also analyzed to help confirm diagnosis and control for contamination from exogenous DNA sources. Here, there was one embryo diagnosed as affected (A), two embryos that were diagnosed as carriers of the maternal HBB mutation (C and D), and one embryo that was diagnosed as a carrier of the paternal HBB mutation (B). The mutation and SNP calls can be seen in Table 2. The same 4 embryos were also tested for aneuploidy across all 24 chromosomes with 3 euploid embryos (B, C, and D), and one embryo was aneuploid (C-trisomy 9). In this case, embryo B was chosen for transfer and resulted in a singleton pregnancy. The PGT-A results can be seen in Fig. 3. Amniocentesis was performed on the resulting pregnancy and showed a normal female result with the correct mutation call at the HBB locus as well.

In total for the two cases, 12 embryos were biopsied with 11 embryos ultimately analyzed for both mutations in the HBB gene and aneuploidy across all 24 chromosomes simultaneously in a single-tube reaction with one set of bar codes and one sequencing run. There were 7 euploid embryos and 4 aneuploid embryos across both cases. For case 1, there were 2 embryos diagnosed as affected, three embryos diagnosed as carriers (2 paternal mutation, 1 maternal mutation), and two embryos diagnosed as unaffected. For case 2, there was one embryo diagnosed as affected and three embryos diagnosed as carriers (2 maternal mutation, 1 paternal mutation).

A further nine PGT-M cases have been completed by the laboratory; however, embryo transfer has not taken place in these cases to date. A detailed summary of these nine cases can be found in Table 3. Mutations for the additional cases include CD17 CD26, CD41/42, and CD71/72. Briefly, an additional 39 embryos were biopsied and tested for HBB status using the above detailed PGT-M strategy with TSE with all 39 embryos (100%) returning a result for HBB. Overall, there were 8 unaffected embryos, 11 affected embryos, 12 embryos that were carriers for the maternal mutation, and 8 embryos that were carriers of the paternal mutation. For these nine cases, five of the embryo cohorts were also tested for aneuploidy using simultaneous PGT-A testing. Overall, 25 embryos were tested for chromosome aneuploidy with 24 embryos returning a result (96%). There were 2 aneuploid (8.3%) embryos and 22 (91.7%) euploid embryos.

**Table 3:** Additional cases of PGT-M/A without clinical outcome data

Case #	Mutation(s)	Embryos Bx.	Embryos Dx.	# Emb. unaffected	# Emb. affected	No. embryos carrier (M)	# Embryos carrier (P)	# Emb. aneuploid	# Emb. euploid
1	CD26	4	4	1	1	0	2	NA	NA
2	CD26/CD71-72	4	4	1	1	2	0	0	4
3	CD26/CD41-42	6	6	0	2	3	1	0	6
4	Del/CD17	4	4	1	0	2	1	NA	NA
5	CD17	8	8	4	3	1	0	0	8
6	CD26	3	3	0	2	0	1	1	2
7	CD26	3	3	1	0	2	0	NA	NA
8*	CD26	4	4	0	1	1	2	1	2
9	CD17/CD26	3	3	0	1	1	1	NA	NA
Totals		39	39	8	11	12	8	2	22

Legend: 9 cases of PGT-M and/or PGT-A with laboratory results. NA = PGT-A testing not performed. * = 1 embryo unable to be diagnosed for PGT-A status due to high levels of noise in this sample

## Discussion

Here we report on the combined testing of a single gene defect (beta thalassemia-HBB) and preimplantation genetic testing for aneuploidy (PGT-A) in a single tube with a single sequencing run. Two patients have completed the entire process including transfer of a single embryo for each patient resulting in two singleton pregnancies free of beta thalassemia and chromosomally euploid live births.

In a poster presented at the PGD-IS meeting in Bangkok, Thailand, Warren and colleagues showed the benefit of the TSE protocol compared to conventional PCR methods and spiking primers into the WGA [20]. Here they showed that the TSE protocol was able to diagnose mutations in the BRCA gene along with STR markers without ADO and with very little preferential amplification. Conventional PCR and spiking both suffered ADO and PA in the mutation detection and in the linked markers. While the TSE protocol is not necessarily “universal” like Karyomapping (Vitrolife) and One PGT (Agilent), it offers benefits over these systems in combining aneuploidy detection in a high-throughput, next-generation sequencing system and allows for the direct assessment of mutation(s) in the embryos instead of inferring inheritance through pure linkage analysis.

In all, eleven embryos were tested in the cases with clinical outcomes. We were able to diagnose ten (90.9%) of these eleven embryos for both beta thalassemia mutations and linked SNP markers along with 24 chromosome aneuploidy testing simultaneously. Overall for the HBB mutation, there were three affected embryos, four carriers of the female mutation, two carriers of the male mutation, and two unaffected embryos. For aneuploidy testing, there were seven euploid embryos and four aneuploid embryos including one mosaic embryo (+ 4p).

Beta thalassemia impacts a large proportion of patients in Asia, so having a reliable test combining single gene defect testing and aneuploidy detection is a true step forward for the region. By combining both tests into one system including just a single whole genome amplification and polymerase chain reaction allows for robust and streamlined testing across multiple patients. No matter the reason for undergoing IVF, a certain proportion of embryos are destined to be chromosomally abnormal. While patients at risk for beta thalassemia are thought to be mostly fertile, a certain number of their embryos will not implant due to aneuploidy. A robust, combined test for both single gene defects and aneuploidy should be a step forward for other populations and carriers of other single gene defects.

We eagerly await the clinical results of the further nine patients that have been taken through the system. Our plan is to continue to offer this service to patients and report further clinical data as it becomes available.

## Data Availability

All data from this work has been detailed in the manuscript.
